# Fascicular Injury in True Neurogenic Thoracic Outlet Syndrome Associated with Manual and Shockwave Therapies: A Case Report

**DOI:** 10.1055/a-2700-4984

**Published:** 2025-10-07

**Authors:** Jae Jun Nam, Yeongyoon Koh, Jong Woong Park, In Cheul Choi

**Affiliations:** 1 Department of Orthopedic Surgery, Korea University Anam Hospital, Seoul, Republic of Korea; 2 Christine M. Kleinert Institute for Hand and Microsurgery, University of Louisville, Louisville, Kentucky, United States

**Keywords:** true nTOS, brachial plexus, fascicular rupture, cervical rib, lower trunk injury

## Abstract

True neurogenic thoracic outlet syndrome (nTOS) is a rare condition resulting from brachial plexus compression, frequently associated with congenital anomalies such as cervical ribs or fibrous bands. We report a unique case of nTOS involving both a cervical rib and fibrous band, complicated by an incidental intraoperative finding of a fascicular rupture in the lower part of lower trunk. Notably, the patient had no history of trauma but had undergone multiple sessions of manual therapy and a single extracorporeal shockwave therapy to the cervical region, raising concerns about a potential iatrogenic contribution.

## Introduction

Thoracic outlet syndrome (TOS) results from compression of the brachial plexus, subclavian artery, or vein at the thoracic outlet. Neurogenic TOS (nTOS) is the most common subtype and involves compression of brachial plexus, whereas venous (vTOS) and arterial (aTOS) types involve the subclavian vein and artery, respectively; aTOS accounts for less than 1% of cases. 1 nTOS is further categorized as either “true” (with objective findings) or “disputed,” which comprises 95 to 99% of nTOS cases. 1 2 Contributing factors include soft tissue anomalies such as scalene muscle hypertrophy, 3 aberrant muscle insertions, 4 accessory scalenus minimus, tumors, 5 and osseous anomalies like cervical ribs, 6 elongated C7 transverse processes, 7 secondary structural changes following trauma such as first rib or clavicle fractures, 8 9 joint disruptions, 10 or tumors. 5

While TOS is classically associated with congenital or traumatic structural causes, therapeutic interventions involving mechanical forces, such as extracorporeal shockwave therapy (ESWT) and manual therapies, may also pose risks to nearby neurovascular structures. 11 Here, we present a unique case of true nTOS with Type III cervical rib with an incidental intraoperative finding of a fascicular rupture in a patient who had undergone both manual therapy and ESWT to the cervical region.

## Case Report

**Video 1** High-resolution ultrasonography demonstrating irritation of the lower trunk and medial cord.


A 48-year-old woman with a history of Moyamoya disease presented with chronic right cervical neck pain and progressive right-hand weakness over a 5-month period. Her initial symptoms included thumb weakness, which gradually progressed to impaired fine motor function, such as tying shoelaces and buttoning clothes. She denied any history of trauma but reported undergoing multiple sessions of manual therapy and a single ESWT targeting the cervical region. Her symptoms worsened following these interventions, prompting referral to a tertiary center.

**Fig. 1:**
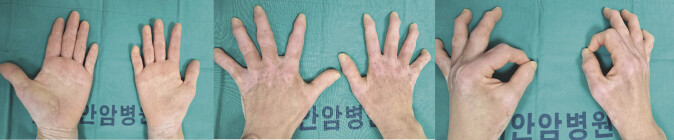
Clinical presentation of neurogenic thoracic outlet syndrome. Visible atrophy of the right first dorsal interosseous, adductor digiti minimi, and thenar muscles, consistent with lower trunk brachial plexopathy.

On examination, the patient demonstrated Grade 2 grip strength and notable atrophy of the thenar eminence, first dorsal interosseous, and adductor digiti minimi muscles ( Fig. 1 ). Tenderness was elicited over the right cervical region, and multiple provocative maneuvers for thoracic outlet syndrome (TOS) yielded positive results.

**Fig. 2:**
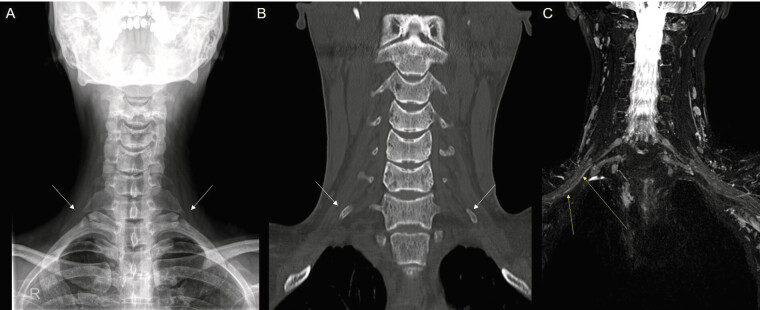
Radiological evaluation of brachial plexus involvement. ( **A** ) Plain radiogram showing bilateral cervical ribs (white arrows). ( **B** ) CT scan showing bilateral cervical ribs (white arrows). ( **C** ) MRI of the brachial plexus, revealing increased T2 signal intensity in the right inferior trunk, medial cord, and distal branches (yellow arrows). CT, computed tomography; MRI, magnetic resonance imaging.

Imaging revealed bilateral cervical ribs and a right-sided costovertebral spur ( Fig. 2A, B ). High-resolution ultrasonography demonstrated irritation of the right lower trunk and medial cord ( Video 1 , available in online version only). Magnetic resonance imaging showed increased T2 signal intensity along the right inferior trunk, medial cord, and distal branches, suggesting neural inflammation ( Fig. 2C ). Computed tomography angiography confirmed vascular patency, effectively ruling out aTOS or vTOS. Electrodiagnostic studies were consistent with an incomplete brachial plexopathy involving the lower trunk, supporting the diagnosis of nTOS.

Given the patient's rapid neurological decline and the presence of structural anomalies, surgery was undertaken. A supraclavicular approach was employed to access the thoracic outlet. The platysma, omohyoid, and deep cervical fascia were dissected, with careful preservation of the external jugular vein, supraclavicular nerves, and phrenic nerve. The scalene fat pad was mobilized to expose the brachial plexus.

**Fig. 3:**
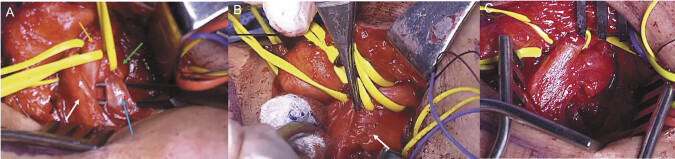
Intraoperative findings during cervical rib resection and scalenectomy. ( **A** ) Intraoperative view demonstrating the brachial plexus components within the supraclavicular fossa. The upper trunk (white arrow), middle trunk (orange arrow), and lower trunk (green arrow) are clearly identified. A fibrous cervicothoracic band (blue arrow) is visualized coursing beneath the lower trunk, exerting compressive force. ( **B** ) Following resection of the cervical rib and associated fibrous band, a partial fascicular rupture of the lower portion of the lower trunk (white arrow) is incidentally discovered, indicating localized neural injury likely exacerbated by chronic mechanical compression. ( **C** ) Intraoperative image showing the microsurgical epineural repair of the disrupted fascicles using fine suture technique, performed under high magnification to preserve the integrity and alignment of the nerve architecture.

A fibrous cervicothoracic band originating from a Type III cervical rib was identified compressing the lower trunk ( Fig. 3A ). Following resection of the rib and associated band, a partial fascicular rupture of the lower part of the lower trunk was incidentally discovered ( Fig. 3B ). Epineural repair was performed with microsurgical technique ( Fig. 3C ).

At 3-month follow-up, the patient reported marked resolution of cervical pain and gradual improvement in hand strength and function.

## Discussion

This case presents a rare and clinically significant finding of fascicular rupture of lower part of lower trunk in a patient with nTOS, following a series of manual therapies and extracorporeal ESWT treatments to the cervical region. To the best of our knowledge, this is the first reported case suggesting a potential association between manual therapy and ESWT and direct brachial plexus injury at the root level, particularly in the setting of anatomical predispositions such as a cervical rib and fibrous band.

Cervical ribs are a well-known cause of TOS and are classified into four types (Type I–IV) based on their degree of development and articulation. 12 Type III cervical ribs, such as in this case, consist of a partially ossified rib with a fibrous band extending toward the first rib. These fibrous bands are significant because they can tether or compress adjacent neurovascular structures, particularly the lower trunk of the brachial plexus. 6 This structural anomaly likely contributed to chronic mechanical irritation and increased the susceptibility of the fascicular rupture to attritional injury. The narrowed costoclavicular space and associated fibrous band may have predisposed the nerve root to repeated microtrauma, even in the absence of overt physical trauma.

The patient had undergone multiple sessions of manual therapy to the neck. Although often beneficial, cervical manual therapies—including high-velocity thrusts, deep tissue mobilization, and passive stretching—have been implicated in rare but serious neurological complications. These include vertebral artery dissection, 13 cervical radiculopathy, and brachial plexus injuries, 11 14 especially in patients with unrecognized congenital or structural vulnerabilities. Reports of brachial plexus strain and neuropathy following aggressive or improperly targeted manual techniques further support the possibility that repeated manipulation could have compounded stress on an already compromised lower trunk of the brachial plexus. 11 Furthermore, while ESWT has gained wide acceptance as a noninvasive and generally safe modality for treating various musculoskeletal conditions, there is increasing recognition of its potential to cause unintended neurological complications. Documented cases of peripheral nerve injury include ulnar neuropathy following ESWT for medial epicondylitis, 15 and bilateral ulnar nerve injuries after radial ESWT for epicondylitis. 16 In preclinical models, shock waves can induce cavitation, vibrational forces, or transient perineural compression, which have been associated with axonal or myelin damage. 17 These cases underscore the risk of nerve irritation or injury when energy is applied too close to superficial or deep neural structures. However, to date, there have been no published reports implicating ESWT in brachial plexus injury.

This case highlights several important considerations. First, it underscores the need for heightened clinical suspicion for structural anomalies, such as cervical ribs or fibrous bands, in patients presenting with atypical upper extremity symptoms. Second, it calls for greater caution when applying ESWT or manual therapies near the cervical neurovascular structures, especially in patients with unexplained neurological findings. Third, it reinforces the value of timely surgical exploration in patients who fail to respond to conservative management and exhibit signs of lower trunk brachial plexopathy. In this case, surgical decompression not only confirmed the diagnosis of true nTOS but also enabled primary repair of a partial nerve rupture—an intervention that might have been missed with nonoperative management alone.

## Conclusion

This case highlights a rare intraoperative finding of fascicular rupture in the lower trunk of the brachial plexus in a patient with true nTOS and congenital anatomical anomalies. While causality cannot be confirmed, the temporal association with manual therapy and ESWT raises important clinical considerations. Clinicians should be vigilant when applying mechanical therapies near the cervical region, especially in patients with atypical neurological symptoms or structural vulnerabilities.
